# EAAT2 Activation Regulates Glutamate Excitotoxicity and Reduces Impulsivity in a Rodent Model of Parkinson’s Disease

**DOI:** 10.1007/s12035-024-04644-0

**Published:** 2024-12-04

**Authors:** Sanjay Das, Kyle Mccloskey, Binod Nepal, Sandhya Kortagere

**Affiliations:** Department of Microbiology and Immunology, Drexel University College of Medicine, Philadelphia, PA 19129 USA

**Keywords:** 5CSRTT, Cognitive impairment, EAAT2, Impulsivity, NMDA receptor subunits, Parkinson’s disease

## Abstract

Parkinson’s disease (PD) is a systemic disease characterized by motor and nonmotor impairments. Loss of dopaminergic neurons in the substantia nigra pars compacta region in PD disrupts dopamine-glutamate homeostasis in the corticostriatal circuit, contributing to cognitive impairment. In addition, excitatory amino acid transporter-2 (EAAT2), localized predominantly to astrocytes and responsible for > 80% of synaptic glutamate clearance, is downregulated in PD, causing glutamate spillover and excitotoxicity. This altered dopamine-glutamate homeostasis and excitotoxicity may affect reward-mediated decision-making behaviors and promote impulsive behaviors in PD. In this study, we hypothesized that GTS467, a small-molecule activator of EAAT2, could effectively reduce excitotoxicity and treat cognitive impairment without promoting impulsive behavior in PD. Rats that were unilaterally lesioned with the 6-OHDA toxin to produce Parkinsonian symptoms were referred to as lesioned rats. Lesioned rats were trained to meet baseline criteria in a 5-choice serial reaction time task, and the chronic effects of GTS467 were assessed after 3 weeks of treatment. The results showed that chronic treatment with GTS467 significantly improved correct responses and reduced premature impulsive responses and omissions compared with saline treatment. This improvement in performance correlated with a reduction in glutamate levels, an increase in EAAT2 expression, and normalization of NMDA receptor subunit expression and signaling. Furthermore, transcriptomic studies on the prefrontal cortex tissue have shown the differential expression of genes involved in neuroprotection, neuroinflammation, learning, and memory. These results validate the role of glutamate excitotoxicity in promoting impulsive behaviors and suggest that GTS467 can be developed as a therapeutic agent to reduce cognitive impairment and impulsive behaviors in PD.

**Supplementary Information:**

The online version contains supplementary material available at 10.1007/s12035-024-04644-0.

## Introduction

The pathology of Parkinson’s disease is characterized by the loss of dopaminergic (DA) neurons in the substantia nigra pars compacta (SNpc) region of the brain, leading to reduced DA innervation of the striatum, and consequent functional changes in the basal ganglia networks, resulting in the dysregulation of direct and indirect pathways, and importantly, the loss of DA-glutamate homeostasis [1, 2]. These functional changes in the basal ganglia and altered DA-glutamate homeostasis in the striatum contribute significantly to several parkinsonian motor symptoms [3–5]. In addition to classic motor impairment, patients with PD also suffer from non-motor symptoms, such as mild cognitive impairment, sleep dysregulation, and gut dysbiosis, which can precede motor impairment by at least a decade [6–8]. Mild cognitive impairment in PD manifests as impairments in learning and memory, sustained attention, cognitive flexibility, decision-making, and other prefrontal cortex (PFC)-mediated executive functions [9]. Furthermore, PD patients often display compulsive and impulsive behaviors which are commonly exacerbated by dopamine replacement therapies [10]. Impulse control disorders (ICD) are multifarious complications characterized by the loss of inhibitory control over impulses, either in the form of impulsive choice or impulsive action. An impulsive choice is an action that does not estimate possible outcomes, while an impulsive action is a premature response, defined as the inability to control and inhibit the action that was initiated, i.e., repetitive behavior [11, 12]. The frontostriatal circuit, involving the dorsolateral PFC, inferior cortex, pre-supplementary area, and basal ganglia, has been implicated in impulsive action [13–15]. This, in conjunction with dopamine overload in the mesolimbic reward pathways caused by dopamine replacement therapies for motor impairment in PD, may be partly responsible for impulsive choice and action [16–19]. DA dysregulation in these circuits results in the overactivation and disinhibition of glutamatergic neurotransmission, which is known to play a major role in the modulation of voluntary movement in PD [20], although its contribution to ICD is not well understood. Glutamate is an excitatory neurotransmitter involved in fast neurotransmission which is stored in presynaptic vesicles and released in high concentrations (in the millimolar range) into the synapse when neurons are depolarized [21]. The released glutamate activates NMDA and AMPA receptors on postsynaptic neurons and is quickly cleared (in less than 10 ms) from the synapse by the sodium- and calcium-dependent excitatory amino acid transporter-2 (EAAT2) or GLT-1 in rodents. EAAT2 is present in perisynaptic astrocytes, while the glutamate taken up by EAAT2 is converted to glutamine by glutamine synthase, and recycled back into neurons to restore the normal glutamatergic tone [22, 23].

Glutamatergic hyperactivity in the striatum, the main basal ganglia input area, has also been implicated in the pathophysiology of the involuntary movements characteristic of LID. Abnormal corticostriatal signaling and excess synaptic glutamate levels in the striatum contribute significantly to the development of LIDs, as observed in rodent and nonhuman primate models of PD [24–26]. Additionally, abnormalities in spiny neuron excitability and firing, as well as the hyperactivity of glutamate transmission, are early physiological changes that may presage and contribute to neurodegeneration in PD by a) impairing modulation of events mediated by glutamate receptors in the striatum, resulting in a net hyperactive signal from basal ganglia output nuclei; b) promoting excess firing of the subthalamic nucleus (STN) neurons, leading to the excess release of glutamate, and the over-activation of synaptic and extra-synaptic NMDA receptors resulting in an increased influx of Na^+^ and Ca^2^^+^ ions, excitotoxicity, and progressive death of SNpc neurons [27, 28]; and c) triggering glutamate spill over and over activation of the excitatory neurons in the prefrontal cortex leading to exacerbation of cognitive impairment and promoting impulsive behaviors [29]. Thus, the modulation of synaptic glutamate is a viable therapeutic option for treating motor and cognitive impairments in PD. Modulating the firing of the STN via deep brain stimulation has been used clinically to control tremors and reduce dyskinesia with varying levels of success [30–32]. The NMDA antagonist amantadine (Gocovri) is the only FDA-approved therapeutic agent for LID [33], although its utility is limited owing to significant side effects. Other NMDA antagonists and mGluR antagonists, such as memantine, Riluzole, and Foliglurax, have been developed based on the rationale of reducing excitotoxicity; however, memantine is associated with serious psychiatric side effects associated with the blockade of NMDAR activity [34–39].

One alternative to the NMDAR antagonist approach to reduce glutamate excitotoxicity is to enhance the reuptake of glutamate from synapses by activating EAAT2, which is present in the perisynaptic processes of astrocytes, and is closely associated with synapses. EAAT2 is a major glutamate transporter, and is responsible for clearing 80–90% of the synaptic glutamate [23, 40]. Interestingly, among patients with PD and other neurodegenerative diseases such as Amyotrophic Lateral Sclerosis (ALS) and Alzheimer’s disease (AD), EAAT2 is downregulated, leading to the exacerbation of excitotoxicity due to the low clearance of glutamate from the synapse [41–43]. Activation of EAAT2 has been demonstrated as a useful therapeutic option to treat ALS, AD, PD, epilepsy, and stroke in preclinical studies, while several EAAT2 activating compounds have advanced through phase 2 clinical trials for ALS [44–51]. In a recent study, EAAT2 was validated as a therapeutic target for PD through analyses showing that knocking down its expression in astrocytes led to an increase in reactive astrocytes, progressive motor deficits, and nigral DA neuronal death in a mouse model of PD [52]. Furthermore, EAAT2 downregulation was observed in genetic and toxin-based rodent models of PD, indicating the translational validity of the target [53–58]. In the PFC, EAAT2 is localized to both astrocytes and excitatory neurons and participate in glutamate uptake [20, 59]. However, studies on the expression of EAAT2 in the PFC of postmortem brain samples and rodents from various disease models remain inconclusive [60–65]. There are currently no reports on stoichiometric assessments of EAAT2 membrane expression correlating with glutamate uptake levels in the PFC. The lack of correlation has been attributed to the accumulation of EAAT2 on the membrane due to post-translational modifications by ubiquitination enzymes [61]. Although there is a strong rationale for targeting EAAT2 in PD, studies on its role in cognitive impairment have been inconclusive. We recently designed a series of small-molecule activators of EAAT2, and optimized them for improved pharmacokinetic properties and brain penetration [66]. Subsequent testing of these molecules in a Drosophila model of Huntington’s revealed that they rescued motor and cognitive impairments in transgenic HD flies in a dose-dependent manner [67]. In the present study, we tested the effects of GTS467, a lead molecule, on impulsive behavior in a rodent model of PD, using a 5-choice serial reaction time task (5-CSRTT). Following the behavioral assessment, we evaluated the effects of GTS467 on ameliorating excitotoxicity in the PFC and striatum using biochemical assessment of the NMDA receptor and its cognate signaling partners.

## Material and Methods

### Chemicals and Reagents

All chemicals and reagents were purchased from Sigma Aldrich (CA) either directly or through a Thermo Fisher Scientific. GTS467 was synthesized in-house, as previously described [66]. Sterile saline and all surgical chemicals were purchased from McKesson Inc., while isoflurane was purchased from VetEquip.

### Animals

**Fig. 1:**
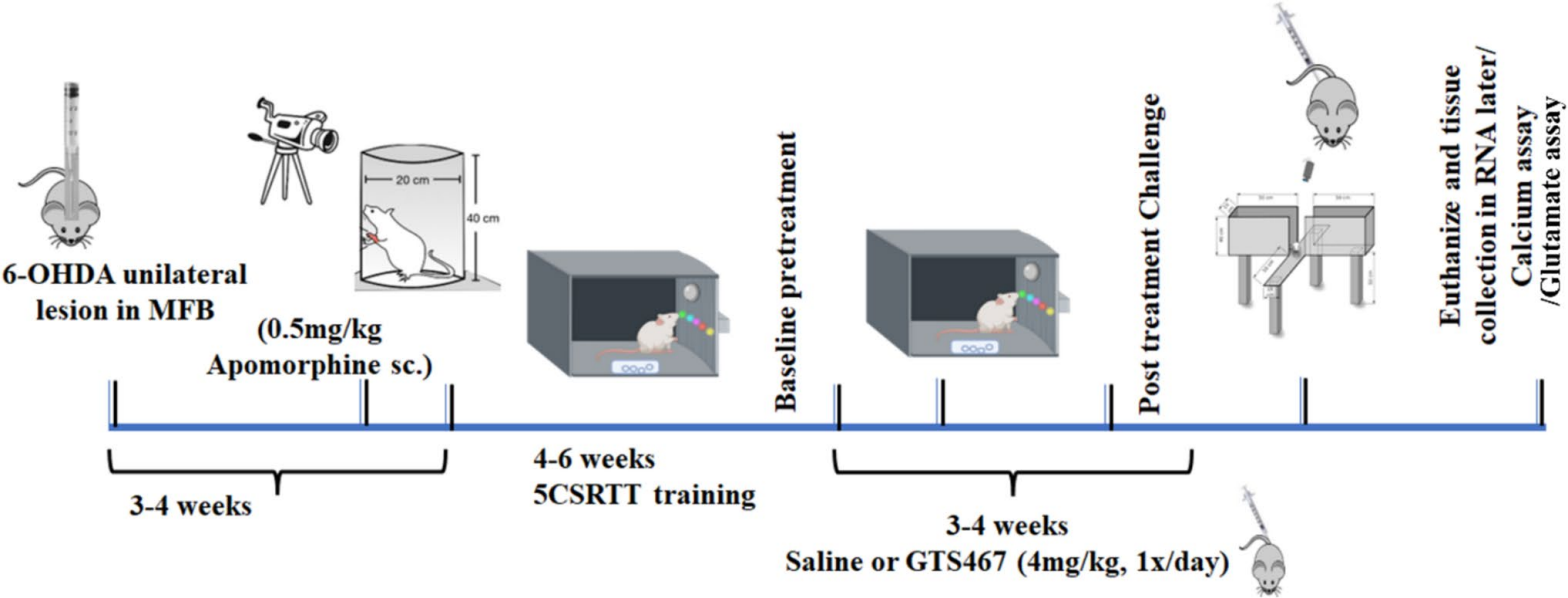
Schematic description of the experimental timeline. Male Sprague–Dawley rats arrive at the facility and get acclimated for 1 week. Rats receive unilateral injection of either 6-OHDA (lesioned group) or saline (sham group) at their MFB and are allowed to recover for 3–4 weeks. At the end of the 4th week, 6-OHDA lesioned rats are administered apomorphine (0.5 mg/kg; s.c) and the number of rotations contralateral to their injection site and ipsilateral rotations are counted in an 1-h-long recording. 6-OHDA lesioned rats are then trained to criteria in a 5CSRTT and their baseline performance is recorded. Lesioned rats are separated into treatment groups and receive saline or GTS467 (4 mg/kg; i.p; 1 × /day for 4 weeks). At the end of weeks, rats are then tested in 5CSRTT behavioral task followed by EPM. Post behavioral tasks, rats are euthanized, and their brains were quickly harvested for biochemical assays

All experiments were conducted in accordance with the National Institutes of Health Guide for the Care and Use of Laboratory Animals, while all procedures were approved by the Institutional Animal Care and Use Committee of Drexel University. Male Sprague–Dawley rats (*n* = 8/gp for behavioral experiments, *n* = 3–5/gp for calcium and glutamate assays, *n* = 3–6/gp for WB, and *n* = 3–4/gp for RNA-Seq study; 275–300 g; Charles River Laboratories) were housed two per cage, and maintained at optimal room temperature and on a 12-h light–dark cycle. Standard rat chow and water were provided ad libitum. The experimental timeline used in this study is presented in Fig. 1. One week prior to the start of behavioral testing, the rats were maintained on a food restriction schedule (maintaining their body weight at 80% of their baseline value) that lasted until the last day of behavioral testing. Rats also received sugar pellets as a food reward during behavioral testing and were fed regular chow one hour post behavioral training/testing sessions.

### Surgery for Generating PD Phenotype

Surgeries were performed as previously described [68]. In brief, to generate lesioned rats, 6-hydroxydopamine (6-OHDA. HBr) (4μL of 10 mg/ml solution dissolved in 0.9% saline and 1% L-ascorbic acid) was injected into the medial forebrain bundle (MFB; coordinates AP: − 2.2 mm, ML: ± 1.5 mm, DV: − 8.0 mm relative to Bregma), at a rate of 1 μl/min, under isoflurane anesthesia, while sham rats were injected with vehicle (6μL of 0.9% saline and 1% L-ascorbic acid) in the same coordinates. The syringe was maintained in place for 4 min to allow the toxin to slowly dissipate into the tissue before it was removed and the incision was sutured. Post-surgery, rats were administered a subcutaneous injection of bupivacaine (0.05 mg/kg) and subcutaneous saline (0.9%) and allowed to recover for 2 weeks. To address site bias, 50% of the rats were lesioned with 6-OHDA on the left side and the other 50% on the right side. Each treatment group had equal distributions of left- and right-lesioned rats.

### Behavioral Task

#### Apomorphine Induced Rotation Task

The rotation task was performed as previously described [68, 69]. In brief, rats were administered a subcutaneous injection of apomorphine (0.50 mg/kg) prior to being placed in a customized Plexiglas cylinder (40 cm H × 30 cm D), after which their locomotor activity was recorded for 30 min. The number of complete rotations contralateral to the side of the lesion was scored by individuals who were blinded to the treatment. Rats were considered to have an effective 6-OHDA lesion if they exhibited ≥ 60 contralateral rotations, with minimal ipsilateral rotations.

#### 5-Choice Serial Reaction Time Task (5-CSRTT) to Measure Attention and Impulsivity

The 5-CSRTT is an operant task measuring sustained attention and impulsivity in rats. When a trial was initiated, a stimulus was presented at any of the five apertures in a pseudorandom order, while a well-trained rat responded to the stimulus with a nose poke at the correct aperture to receive a food reward. During this trial, if a rat responded correctly to the light stimulus during the fixed time period (limited hold), the rat received a food reward, and the response is recorded as “correct response or CR.” If the rat chose an incorrect aperture, it was classified as “incorrect response or IR” and if the rat did not perform the task within the limited hold, it was classified as an “omission or OM.” Both IRs and OMs lead to a 5 s timeout session featuring no signal, house lights, or rewards. The trial was subsequently reset after a fixed time interval called the inter-trial interval (ITI), during which the rat remained in the operant chamber with a house light. Any rat nose pokes made during the ITI were classified as “premature responses or PMR,” and were used as a measure of impulsivity. In these experiments, a set time of 10 s was used for ITI, and the Correct response (CR), Incorrect response (IR), premature response (PMR), and omissions (OM) were measured during a 30 min or 90 trials assay. The rats were trained to meet the baseline performance criteria (Supplementary Table 1). Rats were subsequently split into two treatment groups (*n* = 8/gp) and treated with either vehicle (saline) or GTS467 (4 mg/kg; i.p;1 × /day) for 3 weeks. Their performance in the 5CSRTT was measured on the challenge day on the last day of the treatment 15 min after drug injection. The data were analyzed using Graphpad (ver10), while an unpaired t-test was performed with statistical significance set at *P* ≤ 0.05.

#### Elevated Plus Maze to Measure Anxiety-Like Behavior

Lesioned rats were injected with either 0.9% saline or 4 mg/kg GTS467, placed in the middle of the elevated plus maze (EPM) 10 min post-injection, and their behavior was recorded for 5 min. The time spent, distance traversed, and frequency of entering the closed and open arms were compared between the vehicle and GTS467 drug treatments. Rats that spent more time in the closed arm, and less time in the open arm were considered to exhibit higher levels of anxiety behavior.

### Biochemical Assays

The behavioral tasks, rats were humanely euthanized, their brains were rapidly dissected on ice, and the substantia nigra (SN), striatum (STR), and prefrontal cortex (PFC) were isolated for various biochemical experiments.

### Western Blot

Proteins were extracted from the PFC, SN, and STR to prepare synaptosomal membrane and cytoplasmic fractions, as previously described [70–73]. In brief, the tissue was homogenized in ice-cold lysis buffer (1000 ml, 50 mM Tris–HCl, pH 7.4, 1 mM EDTA, and 320 mM sucrose) using a 1 × protease inhibitor cocktail, 1 × phosphatase inhibitor cocktail, and 1 mM PMSF. The homogenate was then centrifuged at 1000 × *g* for 5 min at 4 °C, and the resulting supernatant was recentrifuged at 10,000 × *g* for 20 min at 4 °C to generate the synaptosomal pellet and cytosolic fraction. The synaptosomal pellet was then resuspended in 300 µl lysis buffer for western blot studies. The protein content of the lysates was assayed using the DC Protein Assay Kit II (Bio-Rad). Immunoblotting was subsequently performed by loading 20 μg of total protein/lane on a Novex 4–20% Tris–Glycine Mini Gel for separation (GenScript, Cat# M00656) for 2 h and transferred to a PVDF membrane (Thermo Scientific). A detailed list of antibodies used for immunoblotting, along with their associated dilutions, is provided in Supplementary Table 2. All primary antibodies used were raised in rabbits, and the secondary antibody was peroxidase-conjugated goat anti-rabbit IgG (H + L) (1:5,000, Jackson ImmunoResearch Laboratories, Inc.). Chemiluminescence was detected using a Super Signal West Dura Extended Duration Substrate detection kit (Thermo Scientific, cat # 34,075). Immunoblots were quantified by densitometry using ImageJ software and normalized to vehicle treatment.

### Calcium Assay

To assess the levels of intracellular calcium after saline and GTS467 treatment, the PFC and STR regions from the lesioned side of the brain were isolated and washed in cold PBS. Subsequently, the calcium assay [74] was performed according to the manufacturer’s instructions (ab102505, Abcam, US). Tissues were resuspended in calcium buffer (5 × sample volume) and homogenized on ice. The homogenized tissues were centrifuged for 3 min at 4 °C at 13,000 × *g*. The supernatants were collected, diluted 2–fivefold, and 50 µl of each sample was loaded into the 96 well plate. A chromogenic reagent, including samples containing the sample or calcium standards, was added to each well. Finally, a 60 µl calcium assay buffer was added into each well and mixed properly and incubated at room temperature for 10 min. The absorbance was measured at 575 nm, and the calcium concentration in the test samples was calculated from the standard curve.

### Glutamate Assay

The glutamate assay was performed following the manufacturer’s instructions (ab83389, Abcam, US) from the PFC and STR tissues. In brief, snap-frozen PFC and STR were collected from the lesioned side and washed in cold PBS. The tissues were resuspended in 100 µl of assay buffer, and homogenized on ice. The samples were incubated on ice for 20 min, followed by centrifugation for 3 min at 4 °C at 13,000 × *g*. The supernatants were then collected, and 50 µl of 2 × diluted samples and standards were loaded in duplicates in a 96-well plate. Subsequently 100 µl of reaction mix for each reaction was prepared by adding 90 µl of glutamate assay buffer, 8 µl of developer solution, and 2 µl of glutamate enzyme mix with 92 µl of assay buffer and 8 µl of developer solution used as background mix. Then, 100 µl reaction mix or background mix was added to standard and samples wells or background wells, respectively. The samples were mixed thoroughly and incubated for 30 min at 37 °C. Absorbance was measured at 450 nm, and the glutamate concentration (µM) in the test samples was calculated from the standard curve.

### RNA Sequencing

The PFC and STR from the lesioned hemisphere were collected from lesioned rats post-behavioral testing, and stored in RNA-later until further use. The tissue was processed and purified, RNA was extracted from the samples using TRIzol reagent, and cDNA libraries were prepared using polyA selection. The average RIN score was 6.36 and DV200 was 79.93%. Libraries were sequenced on an Illumina HiSeq instruments with 2X150bp reads. Salmon was used to map and quantify the raw reads against the GRCh38 reference transcriptome [75], which was imported into the R software (R 4.3.1) via tximport [76]. Data were assessed for differential expression (DE) using DESeq2 [77] by correcting for multiple hypothesis testing and controlling the false discovery rate [78]. Transcripts without a HGNC symbols were discarded and the genes which met the criteria defined by padj ≤ 0.05, −1 ≤ log2FC ≥ 1, were used for all further analyses.

### Statistical Analysis

Data are presented as the mean ± SEM and the details of individual statistical tests used are provided in the figure legends. For all western blot experiments, tissues from the PFC and STR were considered as separate independent groups. Comparison of two independent groups was performed using Student’s* t*-test. One-way analysis of variance (ANOVA) followed by multiple comparisons was performed for three or more groups. For all statistical tests, the significance level was set at 0.05.

## Results

### 6-OHDA Toxin Induction Produced Stable Parkinsonian-Like Symptoms in Rats

**Fig. 2:**
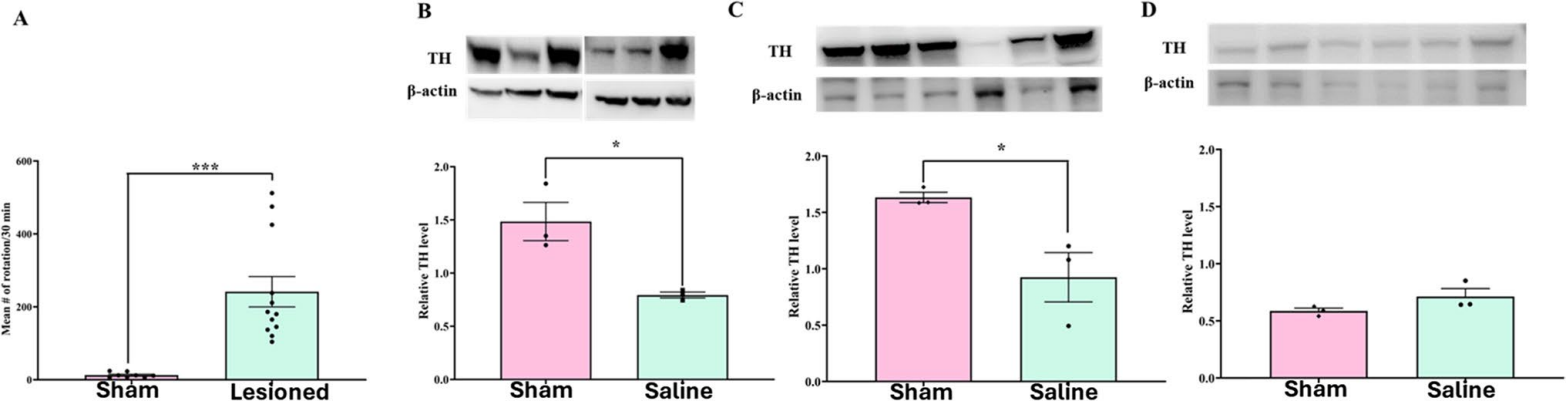
6-OHDA induced unilateral lesion model of Parkinson’s disease shows loss of TH expression (DA biosynthesis) in SN and STR but not in PFC. (**A**) Number of contralateral rotations induced by apomorphine (0.5 mg/kg; s.c) in sham (*n* = 8) and PD (*n* = 12) rats. Unpaired *t*-test showed a significant increase (t18 = 4.443, ***P < 0.001) in rotation in PD group of animals compared to Sham. Representative western blot images and relative expression of tyrosine hydroxylase (TH) protein quantified in sham and lesioned rats treated with saline in (**B**) SN (t4 = 3.794, *p < 0.05, *n* = 3 rats/gp); (**C**) STR (t4 = 3.169, *p < 0.05,) and (**D**) PFC (not significant). Western blots were quantified as relative levels to the control protein β-actin. Data are represented as Mean ± S.E.M and unpaired *t*-test was performed to test the significance

Rats injected with saline (sham group) or 6-OHDA were subjected to apomorphine-induced rotation. Compared to sham rats, 6-OHDA-induced rats showed significantly higher contralateral rotations with minimal ipsilateral rotations (Fig. 2A; t(18) = 4.443; *P* = 0.0003).

### 6-OHDA Toxin Treatment Significantly Reduced TH Immunoreactivity and GABAergic Abundance in PFC and STR

**Fig. 3:**
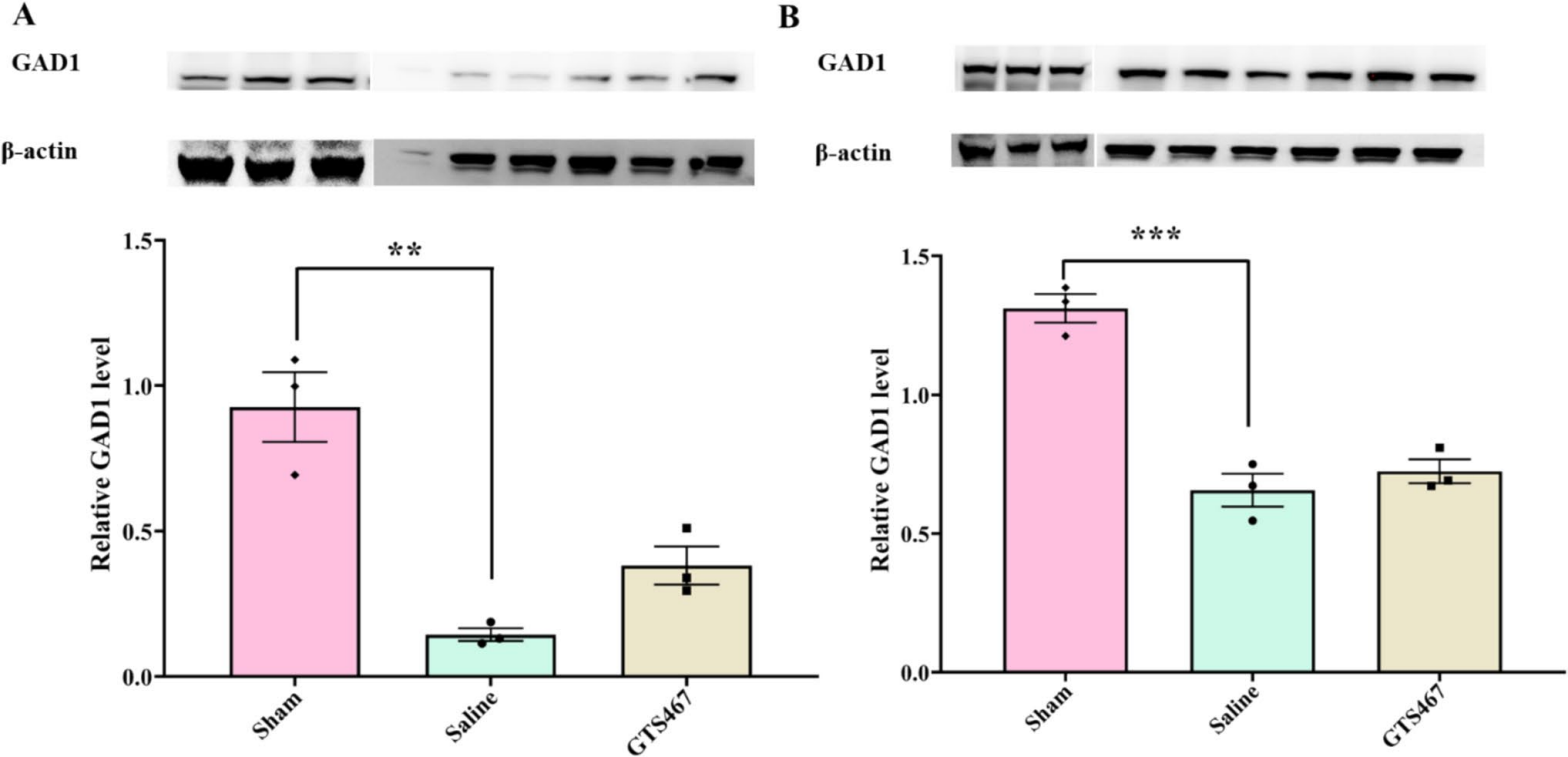
Unilateral lesion model of PD validates reduction in GAD1 levels in PFC and STR (**A**) Representative western blots of GAD1 and β-actin and respective quantified relative levels of GAD1 in sham, lesioned rats treated with saline or GTS467 in (**A**) PFC tissue shows significant reduction in GAD1 levels in lesioned rats compared to sham (F2,6 = 25.34,**p < 0.01, *n* = 3/gp) and a trend towards increase in GAD1 levels with GTS467 treatment (*n* = 3/gp); (**B**) STR tissue shows a significant decrease in relative GAD1 levels in lesioned rats compared to sham (F2,6 = 48.23, ***p < 0.001, *n* = 3/gp) and no change was observed with GTS467 treatment. Western blots were quantified as relative levels to the control protein β-actin. Data are represented as Mean ± S.E.M and one-way ANOVA followed by multiple comparisons were performed to test the significance

Tyrosine hydroxylase (TH) is a rate-limiting enzyme in dopamine biosynthesis that is affected by the loss of DA neurons in the SN. TH expression in the SN, STR, and PFC was measured by western blotting in sham and lesioned rats treated with saline. In the SN and STR, TH expression was significantly reduced in lesioned rats compared to sham rats, while there was no significant change in the PFC (Fig. 2B–D). TH expression was lower in the PFC than that in the SN or STR in both groups. In addition to TH, glutamate decarboxylase (GAD1, also known as GAD67), which catalyzes the conversion of gamma amino butyric acid (GABA) from L-glutamic acid, was reduced in lesioned rats compared to that in sham rats in both the PFC and STR (Fig. 3A and Fig. 3B). Interestingly, chronic treatment with GTS467 led to an increase in GAD1 expression in the PFC, with no change in expression in the STR (Fig. 3).

### GTS467 Treatment Significantly Improved Sustained Attention and Reduced Impulsive Behaviors in Lesioned Rats

**Fig. 4:**
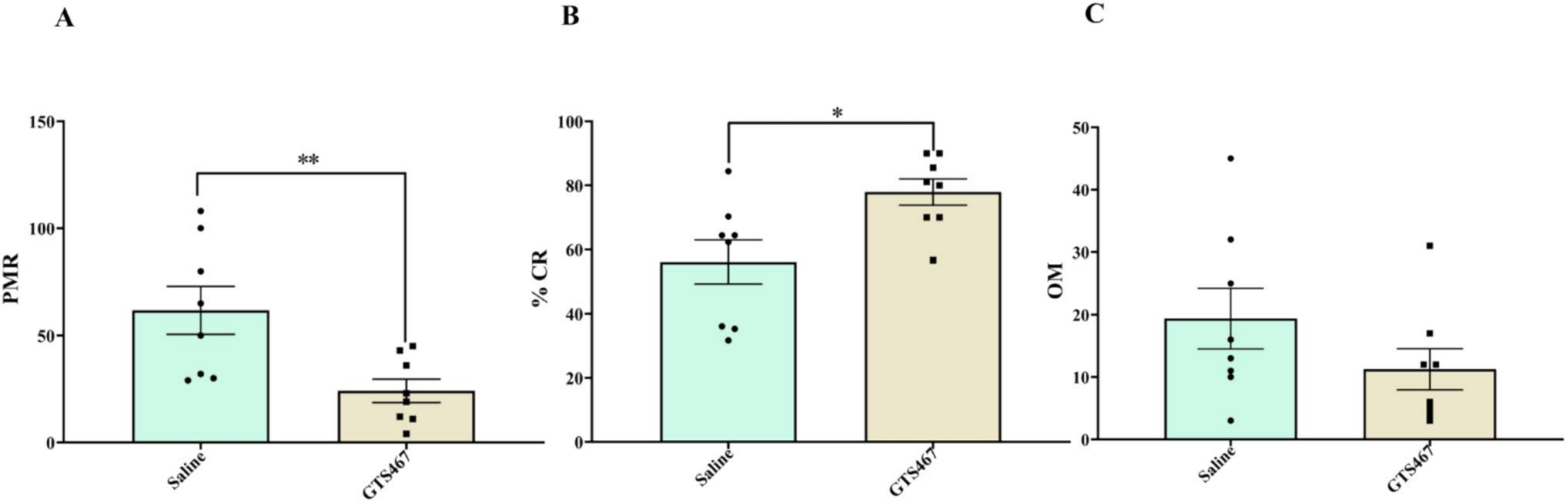
Chronic GTS467 treatment reduced the impulsive response in 6-OHDA-induced lesioned rats. The 5CSRTT task performed by lesioned rats treated with Saline (*n* = 8; i.p; 1x/day for 3 weeks) or GTS467 (*n* = 8; 4mg/kg; i.p; 1 × /day for 3 weeks) showed (**A**) significant reduction in premature response (PMR) (t14 = 3.013, **p < 0.01), (**B**) significant increase in % correct responses (%CR) (t14 = 2.735, *p < 0.05) and (**C**) reduction in omissions (OM) in GTS467-treated rats compared to saline (ns). Data are represented as Mean ± S.E.M and unpaired *t*-test was performed to test the significance

The 5CSRTT task is a goal-directed behavior test that can assess sustained attention and impulsive behavior of lesioned rats. In the present study, chronic treatment with GTS467 (4 mg/kg; i.p; 1 × /day) significantly reduced impulsive behavior in lesioned rats, as determined by the reduction in PMR compared to saline-treated rats (Fig. 4A). In the same rats, GTS467 treatment significantly improved sustained attention in lesioned rats, as measured by the %CR, compared with saline-treated rats (Fig. 4B). This increase in correct performance was associated with a reduction in omissions (OM) in GTS467-treated lesioned rats compared to those in the saline group (Fig. 4C).

### GTS467 Did Not Induce Any Anxiety-Like Behavior in Lesioned Rats

**Fig. 5:**
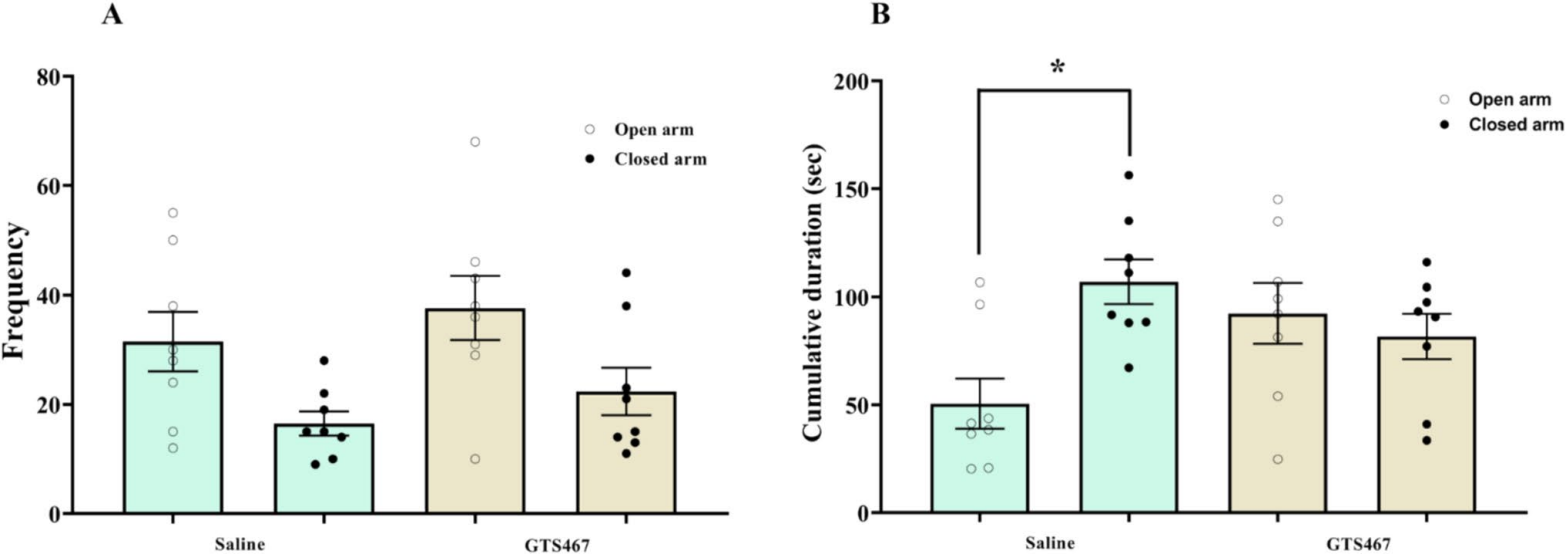
Chronic treatment with GTS467 did not induce anxiety-like behavior in 6-OHDA-induced lesioned rats. Lesioned rats treated with Saline (*n* = 8; i.p) or GTS467 (*n* = 8; 4mg/kg; i.p) were tested on an elevated plus maze for anxious behavior; one-way ANOVA followed by multiple comparison showed no significant change between saline and GTS467 treated rats in (**A**) the frequency and (**B**) significant (*p < 0.01) increase in time spent (s) in the closed arm in saline reflects anxiety whereas no significant changes noticed in GTS467 in open and closed arm of elevated plus maze. Data are represented as Mean ± S.E.M

Lesioned rats treated with either vehicle or GTS467 (4 mg/kg) were assessed for anxiety-like behavior using the EPM. GTS467 treatment did not produce any significant differences in the frequency of entry or time spent in the open and closed arms of the EPM (Fig. 5A, B). However, GTS467 treatment increased the time spent in the open arm, whereas saline-treated rats spent more time in the closed chamber, indicating an increase in anxious behavior (Fig. 5B).

### GTS467 Reduced Intracellular Glutamate Levels by Enhancing Glutamate Uptake by Astrocytes

**Fig. 6:**
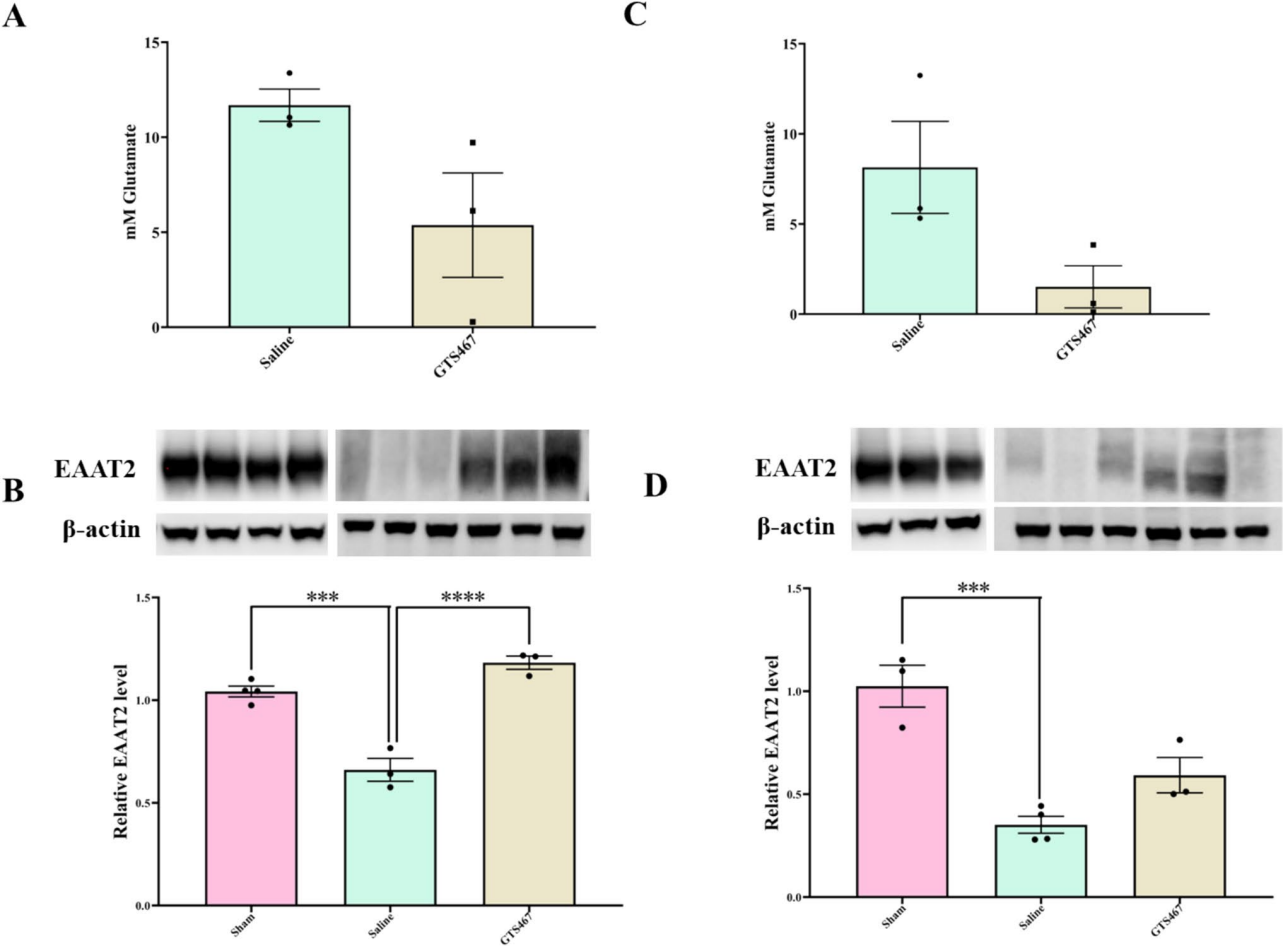
GTS467 treatment normalized glutamate levels with a corresponding increase in EAAT2 expression in PFC and STR of lesioned rats. Lesioned rats treated with GTS467 (*n* = 3; 4mg/kg i.p; 1 × /day for 3 weeks) showed reduction in Glutamate levels compared to saline (*n* = 3; i.p; 1 × /day for 3 weeks) quantified from (**A**) PFC (t4 = 2.194, *P* = 0.0933) and (**C**) STR (t4 = 2.357, *P* = 0.0780). EAAT2 expression was significantly reduced in in lesioned rats compared to sham in (**B**) PFC (F2,7 = 46.32, ***p < 0.001) and (**D**) STR (F2,7 = 21.50, ***p < 0.001) and treatment with GTS467 in lesioned rats led to an increase in EAAT2 expression in PFC (****p < 0.0001).determined using western blots and quantified as relative levels to the control protein β-actin. Data are represented as Mean ± S.E.M and unpaired *t*-test for (A) and (C) and one-way ANOVA with multiple comparison for (B) and (D) was performed to test the significance

GTS467 treatment resulted in a non-significant trend (*P* = 0.093 and *P* = 0.078, respectively) towards decreased intracellular glutamate levels in the PFC and STR tissues (Fig. 6A, C) compared to saline treatment. This reduction in intracellular glutamate levels correlated with a significant increase in the astrocytic glutamate transporter EAAT2 levels in both the PFC and STR of GTS467 treated lesioned rats (Fig. 6B; Fig. 6D). EAAT2 expression was found to be significantly reduced in lesioned rats compared to that in sham rats and was restored upon GTS467 treatment (Fig. 6B; Fig. 6D).

### GTS467 Treatment Alters the Expression of NMDA Subunits in PFC and STR

**Fig. 7:**
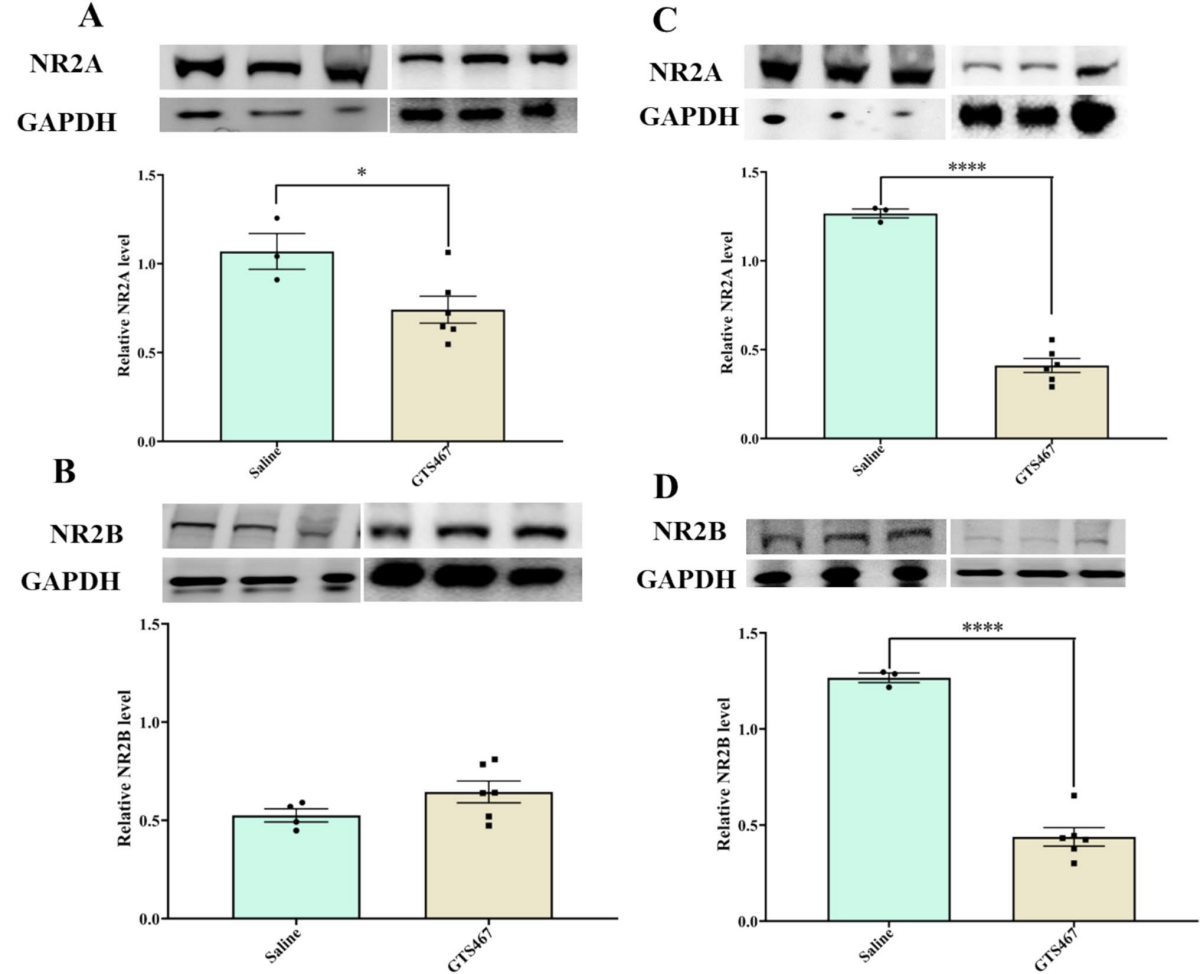
GTS467 treatment reduced excitotoxicity and normalized postsynaptic NMDA receptor subunit expression. Lesioned rats treated with Saline (*n* = 3; i.p; 1 × /day for 3 weeks) or GTS467 (*n* = 6; 4mg/kg i.p; 1 × /day for 3 weeks) showed changes in NMDAR subunit expression levels (**A**) significant reduction in NR2A levels in PFC (t7 = 2.537, *p < 0.05) and (**C**) STR (t7 = 14.33, ****p < 0.0001; (**B**) no changes between saline and GTS467 treated groups for NR2B subunit expression in PFC and (**D**) significant reduction in NR2B subunit levels in STR (t7 = 11.48, ****p < 0.0001) determined using western blots and quantified as relative levels to the control protein β-actin. Data are represented as Mean ± S.E.M and unpaired *t*-test was performed to test the significance

Immunoblotting of tissue lysates from the PFC isolated from lesioned rats treated with GTS467 revealed a significant reduction in NR2A and an increase in NR2B subunits (Fig. 7A and B). In STR tissues, GTS467 treatment resulted in a significant reduction in both NR2A and NR2B subunits (Fig. 7C, D). There was no change in the expression of GluA1 or GluA2 in either the PFC or STR after GTS467 treatment.

### GTS467 Treatment Normalized Calcium Level in PFC of Lesioned Rats

**Fig. 8:**
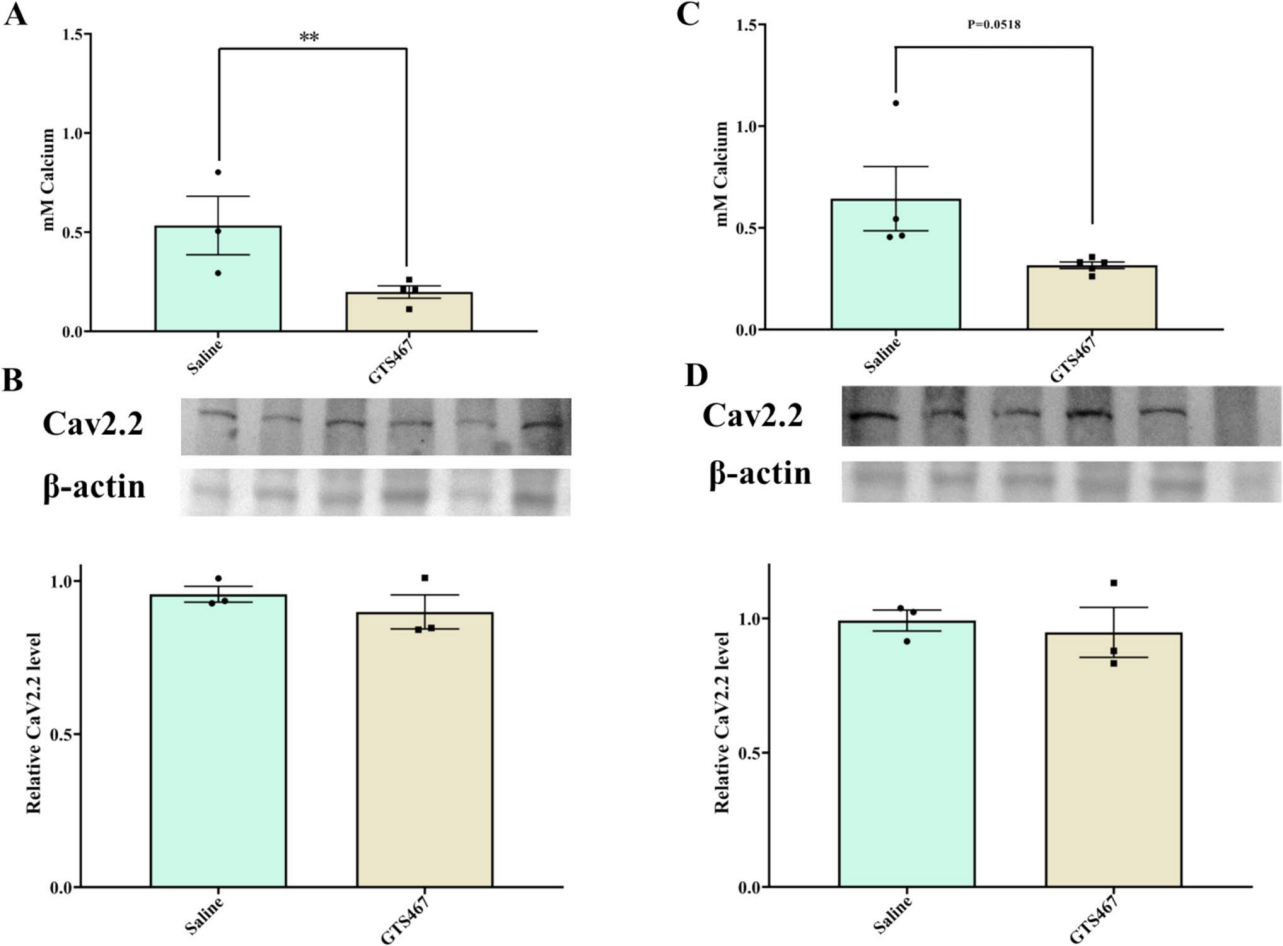
Chronic GTS467 treatment reduced intracellular calcium levels in PFC and STR. Lesioned rats treated with saline (*n* = 3; i.p; 1 × /day for 3 weeks) or GTS467 (*n* = 4; 4mg/kg i.p; 1 × /day for 3 weeks) showed reduction in intracellular calcium levels with GTS467 treatment in (**A**) PFC (t7 = 2.6, **p < 0.05) and (**C**) STR (t7 = 2.341, *P* = 0.051) but had no changes in the relative expression of CAV2.2 in either PFC (**B**) or STR (**D**) determined using western blots and quantified as relative levels to the control protein β-actin. Data are represented as Mean ± S.E.M and unpaired *t*-test was performed to test the significance

Chronic treatment with GTS467 in lesioned rats normalized the excess intracellular calcium levels in the PFC (**p < 0.01) and STR (*P* = 0.0518) tissues compared to saline-treated rats (Fig. 8A, C). This reduction correlated with a trend towards a reduction in the expression of the calcium channel CAV2.2 in the PFC and STR (Fig. 8B, D). However, there was no change in the expression of other calcium-binding proteins, such as calbindin or CAMKIV in the PFC or STR following GTS467 treatment (Supplementary Fig. 1).

### GTS467 Moderately Enhanced the Expression of Neuroprotective Proteins in PFC and STR

**Fig. 9:**
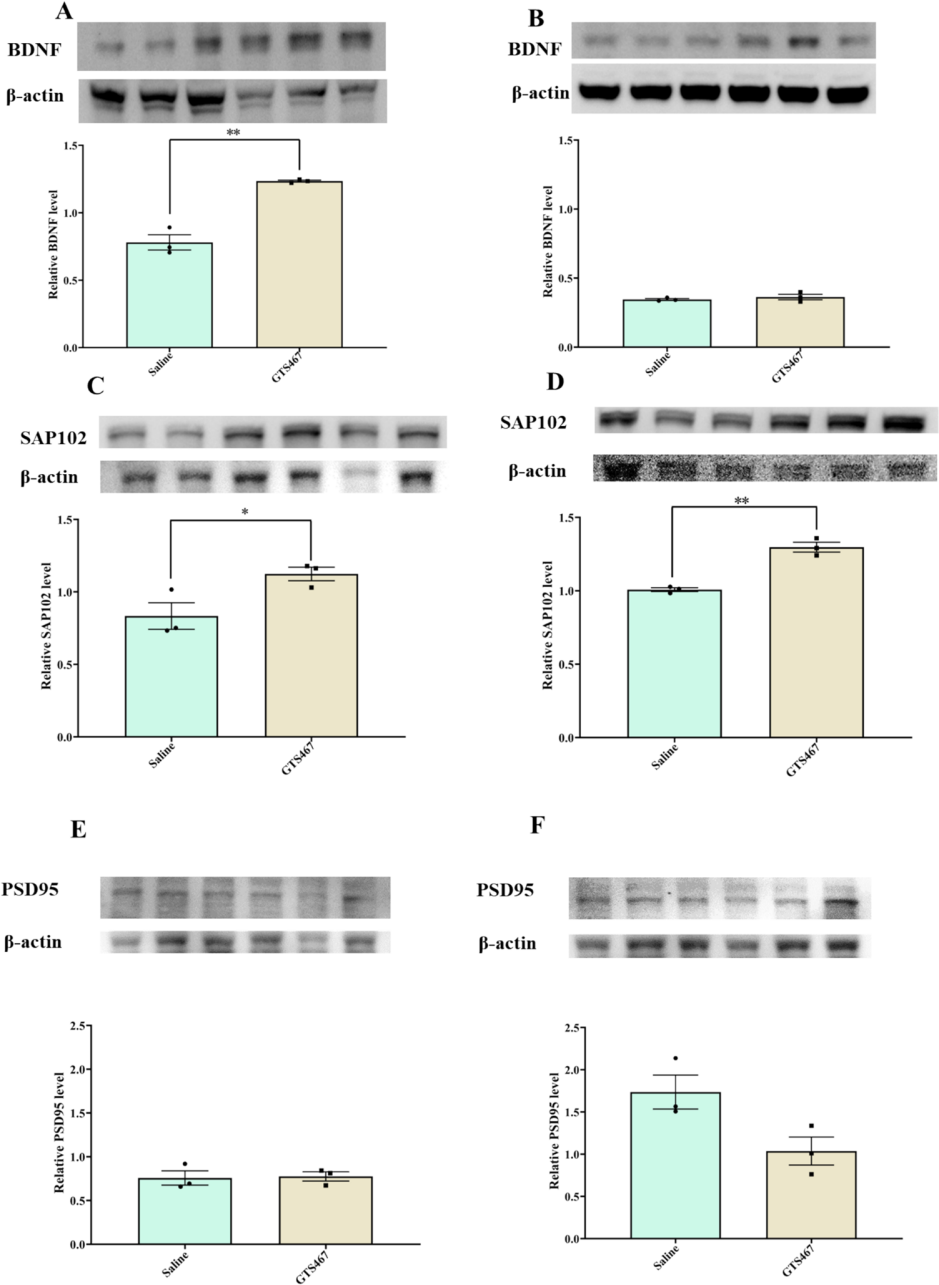
GTS467 treatment regulated intracellular proteins to improve survival response in PFC and STR. Lesioned rats treated with saline (*n* = 3; i.p; 1 × /day for 3 weeks) or GTS467 (*n* = 4; 4mg/kg i.p; 1 × /day for 3 weeks) showed (**A**) significant increase in BDNF levels in PFC with GTS467 treatment (t4 = 7.928, **p < 0.01)and (**B**) no change in STR; significant increase in SAP102 levels in (**C**) PFC (t4 = 2.819 *p < 0.05) and (**D**) STR (t4 = 8.028 **p < 0.01). GTS467 treatment (**E**) had no effect on PSD95 levels in PFC (**F**) reduction in PSD95 levels in STR (t4 = 2.681, *P* = 0.055) determined using western blots and quantified as relative levels to the control protein β-actin. Data are represented as Mean ± S.E.M and unpaired *t*-test was performed to test the significance

The chronic administration of GTS467 moderately enhanced the expression of the neuroprotective proteins BDNF (Fig. 9A and Fig. 9B) and SAP102 (Fig. 9C and Fig. 9D) in lesioned rats. NMDA-interacting protein PSD95 in the PFC did not change following chronic GTS467 treatment (Fig. 9E), but were decreased in the STR (*P* = 0.0551) (Fig. 9F).

### GTS467 Treatment Resulted in Gene Expression Changes in PFC in Lesioned Rats

**Fig. 10:**
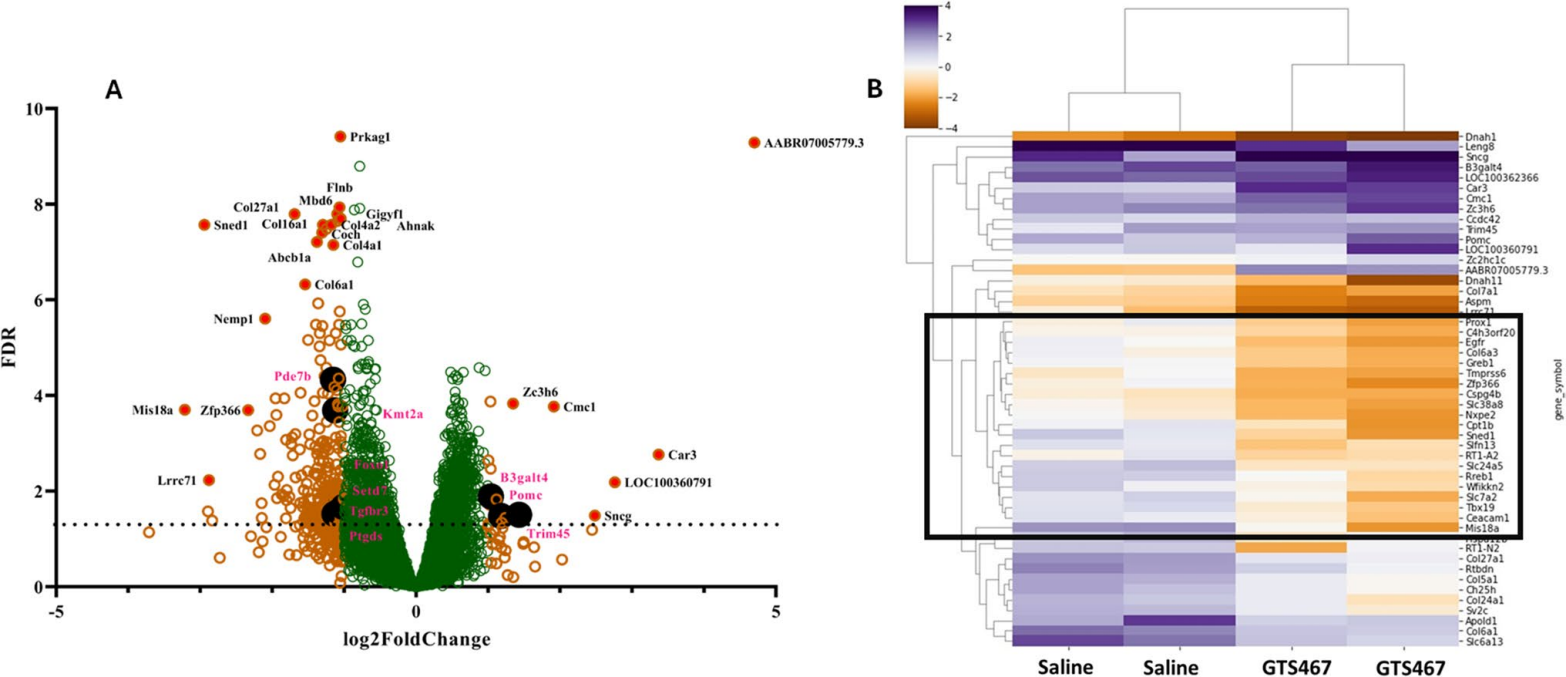
RNA-sequencing revealed differential expression of genes related neuroprotection and cognition. RNA from the PFC of Lesioned rats treated with saline (*n* = 3; i.p; 1x/day for 3 weeks) or GTS467 (*n* = 4; 4mg/kg i.p; 1 × /day for 3 weeks) was sequenced by GeneWiz (Azenta Life Sciences, South Plainfield NJ). (**A**) Mapped reads were analyzed for differential expression using DESeq2 and filtered on Padj < 0.05 and log2FC ≥ 1 or ≤ −1 and plotted as volcano plot with green circles representing genes below the defined cutoff and orange circles representing DEGs with most significantly up- or downregulated genes represented by filled red circles. DEGs represented in black filled circles are involved in conferring neuroprotection or in promoting learning and memory (**B**) Clustered Heat map of top 50 genes sorted by adjusted *p* value. Colored intensity represents scaled transcript abundance. Genes within the black box represent the most significant DEGs that distinguish saline and GTS467 treatment groups

The transcriptomic profiles of the PFC and STR from Saline and GTS467 treated rats were determined using RNA sequencing (RNA-seq). Component analysis of the sample distribution for all four groups revealed a total variance of 70% (PCA1 40%, PCA2 30%). In total, 21,647 differentially expressed genes (DEGs) on different chromosomes were identified in the RNA-seq assay. In the PFC tissue, 8378 genes were upregulated, while 9415 genes were downregulated in GTS467 treated versus saline treated rats. In the STR, 9169 genes were upregulated and 8081 genes were downregulated following chronic GTS467 treatment. A volcano plot of these DEGs identified 249 genes that were significantly up- or downregulated with −1 ≤ log2FC ≥ 1 and Padj ≥ 0.05 with chronic GTS467 treatment (Fig. 10A). Among the 249 significantly up and downregulated genes in the PFC (Supplementary Table 3), nine were found to be directly involved in either the pathogenesis or cognitive impairment in PD. Clustering heatmap analysis of the top 50 DEGs revealed 4–5 subclusters of genes that were highly differentiated between the saline and GTS467 groups (Fig. 10B). These included several genes, transporters, transcription factors, and immune regulator genes that could provide additional insights into glutamate dysfunction in the PFC. In the case of STR, only four genes were found to be significantly up or downregulated between the treatment groups that fit our criteria for significance; hence, the dataset was not further analyzed.

## Discussion

The loss of DA neurons in the SNpc region of the brain in PD leads to several direct and indirect consequences that not only manifest as motor impairment, but also progressive cognitive impairment that affects the quality of life of patients [9]. The loss of DA neurons leads to the dysregulation of several neurotransmitters, including glutamate and GABA, throughout the brain. In the present study, we used a unilateral 6-OHDA induced lesioned rat model to understand the effects of DA loss on the corticostriatal network and its effects on a reward-based 5CSRTT cognitive assay. We hypothesized that the dysregulation of DA-glutamate results in excitotoxicity caused by an excess release of glutamate into the synapse, along with a concomitant reduction in excess synaptic glutamate scavenging by astrocytic EAAT2, which is downregulated in PD, leading to cognitive impairment. GTS467, an EAAT2 activator, was used to test the effects of glutamate restoration on cognitive impairment and evaluate the molecular mechanisms linked to DA-glutamate dysregulation in PD. Results from our study validated the loss of TH expression in the SN and STR, but showed no significant changes in the PFC. Furthermore, our study revealed a significant reduction in GAD1 expression in both the PFC and STR in PD, which was rescued by GTS467 treatment in the PFC, but not in the STR. Previous studies have highlighted the role of GABAergic neurons in the regulation of pyramidal neurons in the dorsolateral PFC during cognitive functions [79–82]. More importantly, the dorsolateral PFC has been implicated in the performance of executive functions such as planning, sequence learning, visual processing, and working memory, which are specifically known to be impaired in PD [83–85]. Studies using in situ hybridization histochemistry in postmortem brain samples from PD patients and healthy individuals revealed a significant reduction in GAD67 (gene encoded by GAD1) mRNA expression in Brodmann area 9 of the prefrontal cortex in PD patients [82]. The molecular mechanisms contributing to the loss of GAD1 in the PFC of patients with PD are poorly understood. Several brain stimulation studies on PD patients with PD have shown that inhibitory pathways in the cortex are less excitable, which may correlate with the loss of GAD1 expression in the cortex [86–88]. In the present study, we found that chronic treatment with GTS467 increased GAD1 protein levels in the PFC, suggesting that excitotoxicity plays a role in the regulation of GAD1 levels.

The PFC, specifically the medial PFC (mPFC), have been implicated in several facets of impulsivity, including decision making [9, 89, 90] and the inability to withhold actions in both motor impulsivity and impulsive choices in humans [91–93] and rodent models [11, 94, 95]. The 5CSRTT is an ideal task for modeling impulsive behaviors in lesioned rats to replicate individual differences in impulsive phenotypes observed in patients with PD [96]. The mPFC is also the center of glutamate signaling, and dysfunction in glutamate regulation in neurological and psychiatric disorders has been shown to be correlated with cognitive impairment [20]. In the present study, we measured impulsivity in lesioned rats, and found that chronic treatment with GTS467 significantly reduced premature responses coding for impulsive behaviors, while increasing their performance in correct choices. To understand the molecular correlates of this behavioral response, we evaluated the expression of key proteins involved in the corticostriatal glutamate pathways. In the PFC, EAAT2 is expressed in astrocytes and excitatory neurons, which could contribute to its ability to regulate extracellular glutamate levels. EAAT2 expression increased with chronic treatment with GTS467 compared to that in the saline-treated groups, which correlated with a reduction in glutamate levels in both the PFC and STR in lesioned rats. While there is some ambiguity in the literature regarding the levels of EAAT2 protein measured in postmortem brain samples, our results concur with published results from preclinical studies [20, 23, 43, 49].

In the mPFC, NMDA receptors play a major role in glutamatergic signaling, while dysfunctional NMDARs have been implicated in PFC-mediated cognitive impairment [55, 97, 98]. More specifically, blocking NMDARs in the PFC or antagonizing the NMDA subunit NR2B results in an increase in motor impulsivity and decision-making. Furthermore, studies have revealed an increased association of the synaptic proteins PSD95 and SAP102 with the NR2B subunit in lesioned rats that exhibited higher levels of motor impulsivity, suggesting that increased expression and function of NMDAR may lead to impulsive behaviors in PD [99–101]. Our results revealed that chronic treatment with GTS467 resulted in a significant decrease in NR2A expression in the PFC and STR, whereas NR2B expression was significantly reduced only in the STR and not in the PFC, compared to saline-treated rats. This decrease in NR2A expression in the PFC and STR influenced the increase in SAP102 expression, while PSD95 showed a moderate decrease in the STR, which is associated with a reduction in NR2B levels in the STR with no change in the PFC. One possibility for the discrepancy observed in our studies is that GTS467 does not regulate NMDAR subunit expression directly, but does so indirectly through the regulation of extracellular glutamate levels. Future studies should test the synergistic effects of NMDAR receptor antagonists and GTS467 in regulating impulsivity in PD.

One consequence of NMDAR-mediated excitotoxicity is increased levels of intracellular calcium, leading to the initiation of pro-death signaling in postsynaptic neurons [102]. We hypothesized that treatment with GTS467 would regulate extracellular glutamate levels to restore normal signaling in postsynaptic neurons. To assess the effects of chronic GTS467 treatment, intracellular calcium levels were measured in the PFC and STR tissues. The results revealed a reduction in intracellular calcium in the PFC and STR, which was positively correlated with the expression of the calcium channel CaV2.2 expression in the PFC and STR of GTS467 treated lesioned rats. CaV2.2 has been shown to be upregulated in PD and plays a major role in regulating intracellular calcium levels in neurons [103, 104]. Our studies also found that BDNF levels were significantly increased in the PFC following GTS467 treatment, indicating a shift towards pro-survival signaling and neuroprotection.

RNA sequencing of PFC and STR tissues from lesioned rats chronically treated with GTS467 revealed differential gene expression compared to saline-treated rats, predominantly in the PFC tissue. Among the upregulated genes in the PFC tissue was B3galt4, which is involved in the synthesis and regulation of GM1 ganglioside in the brain [105, 106], the GM1 ganglioside has been shown to be downregulated in postmortem brain tissue from PD patients [107, 108]. Brain gangliosides have further been shown to exert neuroprotective effects in rodent, non-human primate models of PD [109, 110]. However, the mechanism by which GTS467 upregulates B3galt4 and promotes neuroprotection requires further investigation. Similarly, the gene POMC, which produces proopiomelanocortin, a precursor protein and part of the central melonocortin system, stimulates the production of neuromelanin and endorphins in the SN to protect DA neurons [111] and is upregulated by GTS467 treatment. TRIM45 belongs to the TRIM family of genes that play crucial roles in the turnover of regulatory and misfolded proteins [112]. These genes have been shown to be downregulated in several neurodegenerative diseases, thus leading to the accumulation of misfolded proteins such as α-synuclein [112]. Interestingly, GTS467 treatment led to a modest upregulation of TRIM45, suggesting its role in neuroprotection; however, further studies are needed to confirm and understand the mechanisms underlying this process. Phosphodiesterase (Pde7b), which plays a role in neuroinflammation in PD [113], and KMT2A, a gene involved in the transcriptional dysregulation of genes involved in learning and memory [114], were downregulated by GTS467 treatment in the PFC. These and ~ 20 other genes that were significantly downregulated in PFC tissue from GTS467 treated rats may provide a better understanding of the molecular mechanisms underlying neuroprotection in PD and could be considered as targets for future studies. In this study, we evaluated the molecular effects of GTS467 the systemic dosing only on the lesioned hemisphere of rats. There is significant controversy regarding the role of the contralateral hemispheres in the behavior and function of lesioned models; hence, our future studies will include specific methods to evaluate the circuit-specific effects of DA-glutamate homeostasis on cognitive behavior and their molecular correlates in unilateral and bilateral lesion models of PD.

In conclusion, the loss of DA neurons in the SNc in patients with PD leads to the dysregulation of glutamate neurotransmission, resulting in excitotoxicity that causes excess glutamate release and calcium dysregulation through overactive NMDA receptor signaling, which can accelerate DA neuronal death in the SNc. In the PFC, excitotoxicity caused by glutamate spillover and the overactivation of excitatory neurons exacerbates cognitive impairment, and promotes impulsive behaviors. Recently, we developed GTS467, a novel EAAT2 activator that regulates extracellular glutamate levels, thereby reducing excitotoxicity in patients with PD. In the present study, we demonstrated that chronic GTS467 treatment reduces impulsive behavior and promotes neuroprotection by normalizing glutamate neurotransmission and NMDAR signaling. Transcriptomic analysis of PFC tissue from GTS467 treated lesioned rats suggested additional pathways and mechanisms that may contribute to neuroprotection.

## Supplementary Information

Below is the link to the electronic supplementary material.

Supplementary file1 (DOCX 40 KB)

## Data Availability

Data is provided within the manuscript or supplementary information files.
