# Supplementary material for: EAAT2 Activation Regulates Glutamate Excitotoxicity and Reduces Impulsivity in a Rodent Model of Parkinson’s Disease
Source: Mol Neurobiol. 2024 Dec 4;62(5):5787–803. doi: 10.1007/s12035-024-04644-0 (PMC11953204; doi:10.1007/s12035-024-04644-0)
Supplement: Supplementary file 1 — Supplementary file1 (DOCX 40 KB) [file 12035_2024_4644_MOESM1_ESM.docx]

**Supplementary Table 1:** 5CSRTT training and testing criteria used in this study to evaluate performance determined by the number of correct responses (CR), omissions (OM) with intertrial interval (ITI) set at 10s. Rats were allowed a total of 90 trials in each session and two consecutive days of successful completion of the session under the given criteria led to advancement to next level.

| Program | CR | OM | Total Trial |
| --- | --- | --- | --- |
| RTR8 | (Magazine Training) Total Pellet delivery | | |
| RTR9 | Nose poke training | | |
| RTR1 | >85% | <15% | 90 |
| RTR2 | >85% | <15% | 90 |
| RTR3 | >85% | <15% | 90 |
| RTR4 | >80% | <15% | 90 |
| RTR5 | >70% | <15% | 90 |
| RTR6 | >65% | <15% | 90 |
| RTR7 | >45% | <25% | 90 |
| RTR11 | Testing session | | |

**Supplementary Table 2: Details of antibodies used in this study**

| Sl. NO. | Ab | Dilution | Host | Company | Catalogue# | RRID |
| --- | --- | --- | --- | --- | --- | --- |
| 1 | EAAT2 | (1:1000) | RbmAb | CST | 3838S | AB_2190743 |
| 2 | NR2A | (1:1000) | RbmAb | EMD Milipore | 04-901 | AB_1163481 |
| 3 | NR2B | (1:1000) | RbmAb | Sigma | SAB4300711 |  |
| 4 | CaV2.2 | (1:1000) | RbmAb | CST | 35175 |  |
| 5 | BDNF | (1:1000) | RbmAb | Sigma | SAB2108004 |  |
| 6 | PSD95 | (1:1000) | RbmAb | CST | 3409S | AB_1264242 |
| 7 | Calbindin | (1:1000) | RbmAb | CST | 13176 | AB_2687400 |
| 8 | TH | (1:1000) | RbmAb | CST | 58844 | AB_2744555 |
| 9 | GAD1 | (1:1000) | RbmAb | CST | 41318S |  |
| 10 | CaMKIV | (1:1000) | RbmAb | CST | 4032S | AB_2068389 |
| 11 | SAP102 | (1:1000) | RbmAb | CST | 47421 | AB_2739925 |
| 14 | β-actin | (1:1000) | RbmAb | CST | 4967S | AB_330288 |
| 15 | GAPDH | (1:1000) | RbmAb | CST | 5174 | AB_10622025 |

RbmAb= Rabbit monoclonal antibody, RbPAb= Rabbit polyclonal antibody

**Supplementary Table 3:** List of the differentially expressed genes with Padj <0.05 obtained from RNA-Seq analysis of PFC tissue from saline or GTS467 treated rats.
